# Novel Anti-Microbial Peptide SR-0379 Accelerates Wound Healing via the PI3 Kinase/Akt/mTOR Pathway

**DOI:** 10.1371/journal.pone.0092597

**Published:** 2014-03-27

**Authors:** Hideki Tomioka, Hironori Nakagami, Akiko Tenma, Yoshimi Saito, Toshihiro Kaga, Toshihide Kanamori, Nao Tamura, Kazunori Tomono, Yasufumi Kaneda, Ryuichi Morishita

**Affiliations:** 1 Department of Clinical Gene Therapy, Osaka University Graduate School of Medicine, 2-2 Yamada-oka, Suita, Osaka, Japan; 2 AnGesMG, Inc., 7-7-15 Saito Bio-Incubator, Ibaraki, Osaka, Japan; 3 Division of Vascular Medicine and Epigenetics, Osaka University United Graduate School of Child Development, 2-1 Yamada-oka, Suita, Osaka, Japan; 4 Division of Gene Therapy Science, Osaka University Graduate School of Medicine, School of Child Development, 2-1 Yamada-oka, Suita, Osaka, Japan; 5 Division of Infection Control and Prevention, Graduate School of Medicine, 2-2 Yamada-oka, Suita, Osaka, Japan; Mizoram University, India

## Abstract

We developed a novel cationic antimicrobial peptide, AG30/5C, which demonstrates angiogenic properties similar to those of LL-37 or PR39. However, improvement of its stability and cost efficacy are required for clinical application. Therefore, we examined the metabolites of AG30/5C, which provided the further optimized compound, SR-0379. SR-0379 enhanced the proliferation of human dermal fibroblast cells (NHDFs) via the PI3 kinase-Akt-mTOR pathway through integrin-mediated interactions. Furthermore SR-0379 promoted the tube formation of human umbilical vein endothelial cells (HUVECs) in co-culture with NHDFs. This compound also displays antimicrobial activities against a number of bacteria, including drug-resistant microbes and fungi. We evaluated the effect of SR-0379 in two different would-healing models in rats, the full-thickness defects under a diabetic condition and an acutely infected wound with full-thickness defects and inoculation with *Staphylococcus aureus*. Treatment with SR-0379 significantly accelerated wound healing when compared to fibroblast growth factor 2 (FGF2). The beneficial effects of SR-0379 on wound healing can be explained by enhanced angiogenesis, granulation tissue formation, proliferation of endothelial cells and fibroblasts and antimicrobial activity. These results indicate that SR-0379 may have the potential for drug development in wound repair, even under especially critical colonization conditions.

## Introduction

Antimicrobial peptides are produced by multicellular organisms as a defense mechanism against competing pathogenic microbes [1]. Currently, more than 1,200 antimicrobial peptides have been discovered in animals and plants [2]. In addition to their antimicrobial functions, some peptides, such as LL-37, are known to have other functions. For example, LL-37 is chemotactic for monocytes, T cells, neutrophils and mast cells and also stimulates mast cell histamine release and angiogenesis [3]. These observations have led to the development of a therapeutic concept using antimicrobial peptides as multifunctional effector molecules to prevent infection directly and to promote wound healing in various ulcers. Indeed, many researchers have tried to develop antimicrobial peptides for topical use and systemic application [4] [5]. Before these peptides can be used in clinical applications, several improvements must be made, including 1) increased stability, 2) reduced cytotoxicity and 3) improved antimicrobial activity [6]. To improve the stability of antimicrobial peptides, converting amino acids from L to D conformations has been attempted [7]. Antimicrobial peptides are usually positively charged, between 12 and 100 amino acids in length and form amphipathic structures. The production of shorter analogs may also resolve the cost issue for clinical applications.

We previously developed an antimicrobial peptide named AG30 (angiogenic peptide 30) that contained 30 amino acids and possessed both angiogenic and antibacterial functions [8] [9]. For clinical application in the treatment of drug-resistant ulcers, we have modified AG30 to enhance its angiogenic activity, broaden its antibacterial function, enhance its stability and reduce its cost. In this study, we have demonstrated the production of a stable, shorter peptide, SR-0379, that contains twenty amino acids, including one lysine residue that has been converted to D-lysine. This peptide exhibited antimicrobial activities against a number of bacteria, including drug-resistant bacteria, and induced the proliferation, tube formation, migration and contraction of human dermal fibroblast cells.

Treatment with SR-0379 significantly accelerated wound healing in a skin ulcer model. In the intracellular mechanism of wound healing, we focused on the PI3K/Akt/mTOR pathway. It has been reported that Akt/mTOR activation, by the ablation of Pten and Tsc1, dramatically increased epithelial cell proliferation, migration, and cutaneous wound healing, while pharmacological inhibition of mTOR with rapamycin delays wound closure [10]. Indeed, SR-0379 would activate the Akt/mTOR pathway which lead to accelerate wound healing. Further application of SR-0379 might provide a new therapeutic option to treat various ulcers, such as diabetic ulcers and severe burns.

## Results

### Design of SR-0379

**Figure 1:**
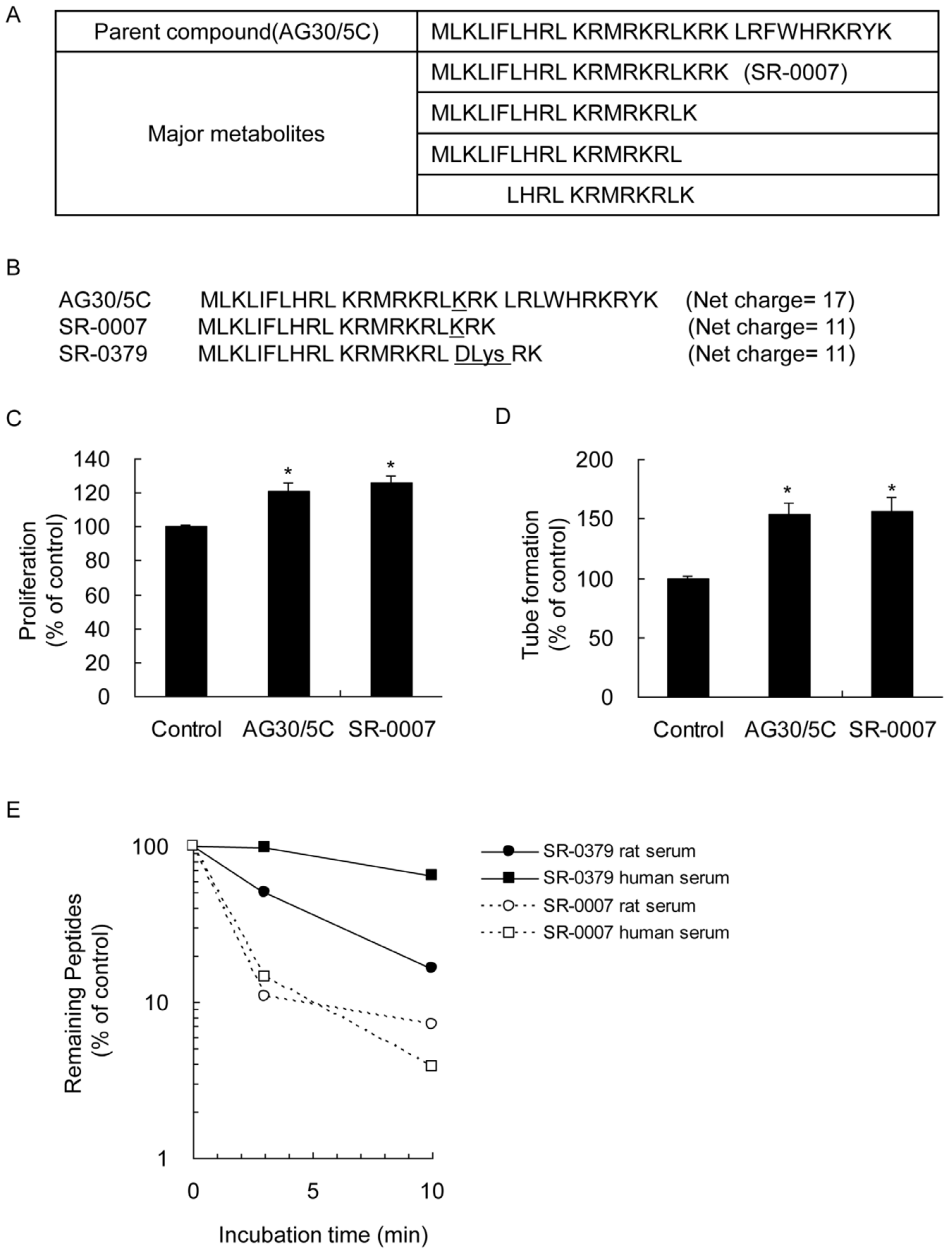
Lead optimization from the angiogenic peptide AG30/5C. A) Major metabolites of AG30/5C determined by MALDI-TOF MS. The parent compound (AG30/5C) was incubated with rat serum *in vitro* 60 minutes. The metabolites were identified by comparison with the pre-incubation peptide. B) Sequences and net charges of AG30/5C and AG30/5C-derived peptides (SR-0007 and SR-0379). The lysine (K) of SR-007 was replaced with D-lysine in SR-0379. C) Effect of AG30/5C (10 μg/ml) and SR-0007 (10 μg/ml) on HUVECs proliferation. N = 3 per group. *P<0.05 vs. control. D) Effect of AG30/5C (10 μg/ml) and SR-0007 (10 μg/ml) on tube formation. The formation of capillary-like structures was observed in co-cultures of HUVECs and NHDFs. N = 5-12 per group. *P<0.05 vs. control. E) Stability of SR-0007 and SR-0379 in rat and human sera. SR-0007 and SR-0379 were quantified before or after incubation *in vitro* with rat and human sera for either 3 or 10 minutes. N = 2.

For use in clinical applications, we first modified AG30 to improve its stability and reduce its cost. To perform the lead-to-drug-candidate optimization, various approaches to improve stability were evaluated. The metabolic stability of AG30/5C was evaluated by using matrix-assisted laser desorption/ionization time-of-flight mass spectrometry (MALDI-TOF MS) (Figure. S1 in File S1). The major metabolites were 20 amino acids (aa), 18 aa and 17 aa, which overlap the N terminus AG30/5C and 12 aa in the middle of AG30/5C (Fig. 1A). The metabolites of AG30/5C were cleaved by endopeptidases and exopeptidase at multiple sites, with most cleavage sites present in the C-terminus. Those metabolites were synthesized (Fig. 1B), and their proliferation in HUVECs, tube formation in co-culture of HUVECs and NHDFs and antibacterial activities against *Escherichia coli*, *Pseudomonas aeruginosa* and *Staphylococcus aureus* were evaluated. Treatment with the 20-aa peptide (SR-0007) yielded significant increases in proliferation of HUVECs (Fig. 1C), whereas other metabolites did not have significant effects (data not shown). The stimulatory effects of SR-0007 were equivalent to the effects of AG30/5C (Fig. 1C). Furthermore, the treatment with SR-0007 (10 μg/ml) induced tube form in co-culture of HUVECs and NHDFs with the same level of AG30/5C (Fig. 1D). SR-0007 exhibited similar antibacterial activities against *Escherichia coli*, *Pseudomonas aeruginosa* and *Staphylococcus aureus* as AG30/5C (Table 1). These results revealed that SR-0007, which consists of 20 amino acids, is a potent candidate metabolite like AG30. However, SR-0007 was rapidly degraded by rat and human sera at 37°C (Figure 1E). Therefore, we further modified SR-0007 to improve its stability. The lysine in SR-0007 was replaced with D-form lysine, and the resulting compound was named SR-0379. As shown in Fig. 1E, the stability of SR-0379 was significantly improved, suggesting that SR-0379 might be resistant to the actions of known natural peptidases [11].

**Table 1:** MICs of several compounds against *E. coli*, *P. aeruginosa* and *S. aureus*.

Compound	*E. coli* (ATCC25922)	*P. aeruginosa* (ATCC27853)	*S. aureus* (ATCC29213)
	MIC (μg/ml)		
AG30/5C	32	16/32	16/32
SR-0007	64	16/32	16/32
SR-0379	8	16/32	16
Tobramycin	0.25–1	0.25–1	0.12–1
Meropenem	0.008–0.06	0.25–1	0.03–0.12
Oxacillin	-	-	0.12–0.5

The scores indicate the MICs (mg/ml) for *E. coli*, *P. aeruginosa and S. aureus*. MICs represent the individual data from two independent experiments.

### Antibacterial activity of SR-0379 in drug-resistant/sensitive strains

We examined the antibacterial effects of AG30/5C, SR-0007 and SR-0379 against *E. coli*, *P. aeruginosa* and *S. aureus* (Table 1). Importantly, SR-0379 exhibited more potent antibacterial activity against *E. coli compared* to the original SR-0007 peptide, whereas the minimal inhibitory concentration (MIC) of SR-0379 against *P. aeruginosa* was equivalent to that of SR-0007. A number of strains were then evaluated for their sensitivities to SR-0379 (Table 2). SR-0379 demonstrated potent antibacterial activity against gram-positive and gram-negative aerobes and anaerobes. Additionally, SR-0379 exhibited antibacterial effects against fungi such as *Candida krusei*. Notably, the antimicrobial spectrum of SR-0379 is broader than the spectra for antibiotics such as chloramphenicol or amphotericin B (Table 2) failed to exhibit an inhibitory activity against fungi or bacteria, respectively. Furthermore, we tested the antibacterial effects of SR-0379 on antibiotic-resistant/sensitive strains for use in clinical applications. Unexpectedly, SR-0379 exhibited similar inhibitory effects on various antibiotic-resistant strains, such as methicillin-resistant *S. aureus* (MRSA) and multidrug-resistant *Acinetobacter baumannii*, compared to the sensitive strains (Table 3). As antimicrobial peptide functional mechanisms are related to the disruption of the bacterial membrane [12], SR-0379 may exhibit potent antibacterial effects against other antibiotic-resistant strains.

**Table 2:** *In vitro* activities of SR-0379 against Gram-positive and Gram-negative bacteria and fungi.

		Gram staining	Strain	MIC (μg/ml)	
				SR-0379	Chloramphenicol
Bacteria	Aerobes	G(+)Bacilli	*Micrococcus luteus*	2	1
			*Bacillus subtilis*	2	4
		G(-)Bacilli	*Salmonella* Enteritidis	8	4
			*Salmonella* Typhimurium	8	4
			*Acinetobacter baumannii*	8	16/32
	Anaerobes	G(-)Bacilli	*Propionibacterium acnes*	16/32	NT
			*Bacteroides fragilis*	32	1
			*Fusobacterium nucleatum*	16/32	0.25
Fungi			Strain	SR-0379	Amphotericin B
			*Penicillium glabrum*	8/16	0.125
			*Fusarium solani*	8	1
			*Alternaria alternata*	32	1
			*Trichophyton mentagrophytes*	32	0.125
			*Trichophyton rubrum*	64	0.125
			*Candida krusei*	32	2

The scores indicate the MICs (mg/ml) for gram-positive and gram-negative bacteria and fungi. MICs represent the individual data from two independent experiments.

NT: Not tested.

**Table 3:** *In vitro* activities of SR-0379 against seven strains of drug-resistant bacteria.

Bacteria	Drug resistance	MIC (μg/ml)
*Pseudomonas aeruginosa*	Aminoglycoside-resistant	16
	Carbapenem-resistant	16–64
	Fluoroquinolone-resistant	16/64
*Staphylococcus aureus*	Methicillin-sensitive	32
	Methicillin-resistant (1)	32
	Methicillin-resistant (2)	32
*Acinetobacter baumannii*	Multidrug-resistant	16

MICs represent the individual data from two independent experiments.

### Cellular functions of SR-0379 and its molecular mechanisms

**Figure 2:**
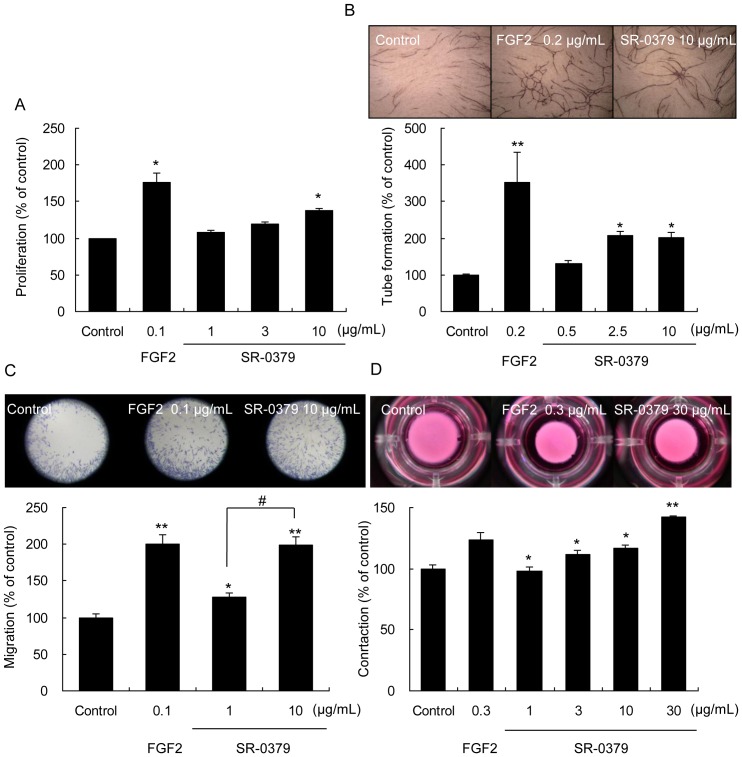
Cellular function of SR-0379. A) Effect of SR-0379 on NHDFs proliferation. NHDFs were treated with SR-0379 (1, 3 and 10 μg/ml) or FGF2 (0.1 μg/ml). N = 4 per group. *P<0.05 vs. control. B) The upper panel shows representative pictures of tube formation in a co-culture of HUVECs and NHDFs (Control, FGF2: 0.2 μg/ml) and SR-0379 (10 μg/ml). The lower panel shows the effects of SR-0379 on tube formation in a co-culture of HUVECs and NHDFs. N = 5 per group. *P<0.05, **P<0.01 vs. control. C) The upper panel shows representative pictures of the migration induced by FGF2 (0.1 μg/ml) and SR-0379 (10 μg/ml). The lower panel shows the effects of FGF2 (0.1 μg/ml) and SR-0379 (1 and 10 μg/ml) on migration. N = 4 per group. *P<0.05, **P<0.01 vs. control, #P<0.01 vs. SR-0379 (1 μg/ml). D) The upper panel shows representative pictures of the fibroblast-collagen-matrix contraction assay with FGF2 (0.3 μg/ml) and SR-0379 (1, 3, 10 and 30 μg/ml). The lower panel shows the effects of FGF2 (0.3 μg/ml) and SR-0379 (1, 3, 10 and 30 μg/ml) on fibroblast-collagen matrix contraction. N = 3 per group. *P<0.05, **P<0.01 vs. control.

We investigated the effects of SR-0379 on the proliferation, migration and contraction capacity of neonatal normal human dermal fibroblasts (NHDFs) and examined the angiogenic activity of HUVECs in co-culture with NHDFs. In the proliferation assay, the treatment with SR-0379 (1, 3 and 10 μg/ml) resulted in significant increase in the proliferation of fibroblasts in a dose-dependent manner (Fig. 2A). Whereas Normal Human Epidermal Keratinocytes (NHEKs) were treated with SR-0379 (1, 3 and 10 μg/ml), SR-0379 did not affect cell proliferation (Figure S2 in File S1). Similarly, in the tube formation assay, the treatment with SR-0379 (0.5, 2.5 and 10 μg/ml) significantly induced tube formation of HUVECs in co-culture with NHDFs as angiogenic activity (Fig. 2B). In the migration assay, the treatment with SR-0379 (1 and 10 μg/ml) also resulted in significant increases in migration activity (Fig. 2C). In the fibroblast-collagen-matrix contraction assay, the treatment with SR-0379 (1, 3, 10 and 30 μg/ml) significantly induced contraction as a measure of wound healing activity (Fig. 2D). When compared to the activities of FGF2 (0.3 μg/ml), the contraction activity of SR-0379 (30 μg/ml) was more potent, while the other activities were equivalent or less potent in SR-0379. Interestingly, the treatment with SR-0379 significantly increased the mRNA expression of interleukin-8 (IL-8) which was attenuated with pretreatment of Wortmannin (PI3kinase inhibitor, 100 nM) (Figure S3A in File S1). Similarly, the treatment with SR-0379 increased IL-8 protein expression in a dose dependent manner Figure S3B in File S1).

**Figure 3:**
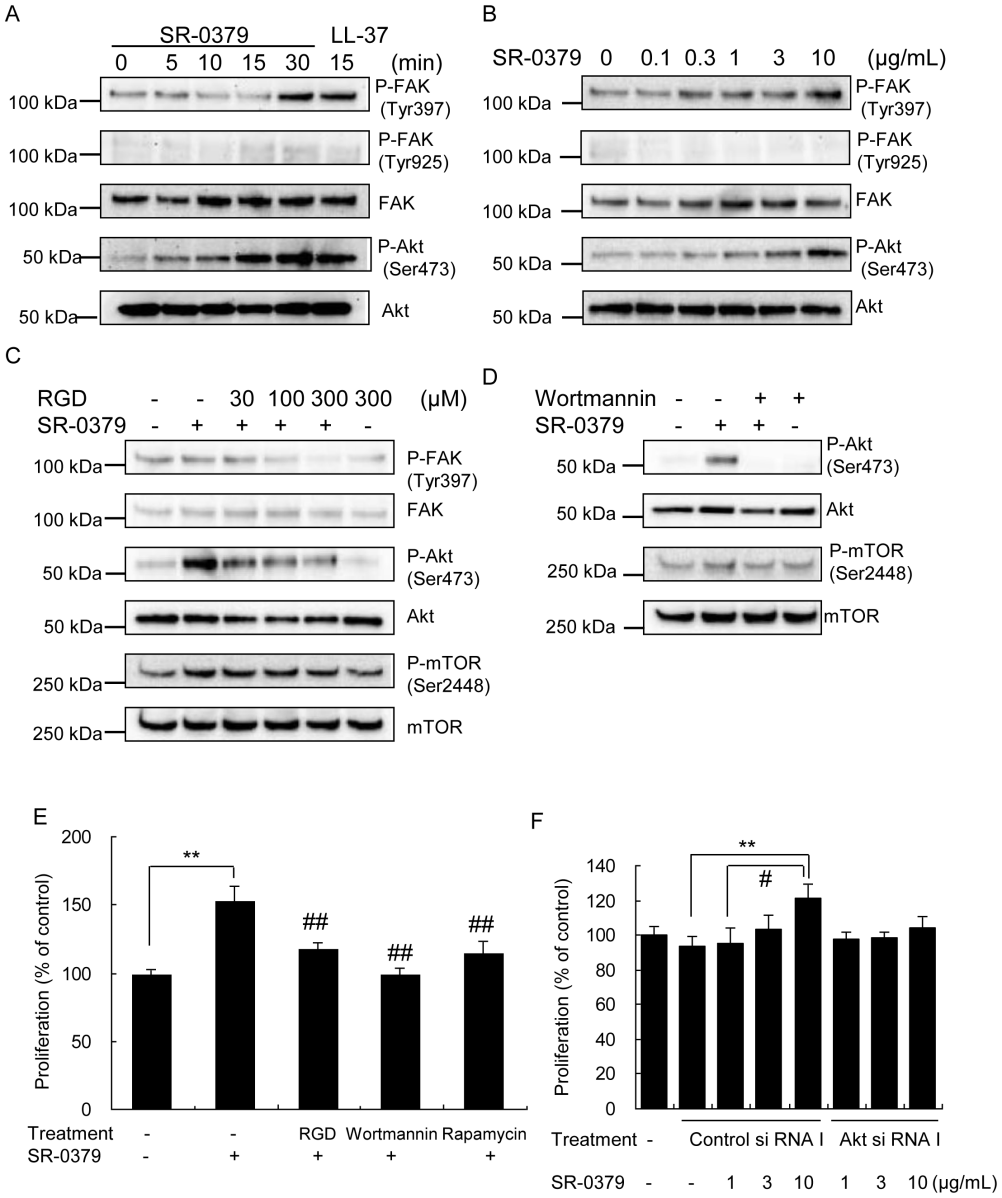
Activation of the PI3 kinase/AKT/mTOR pathway by SR-0379 in NHDFs. A) Effects of SR-0379 on phosphorylated FAK (Tyr397 and Tyr925) and phosphorylated Akt (Ser473) as determined by Western blot. The cells were treated with SR-0379 (10 μg/ml) for 0, 5, 15 and 30 minutes or LL-37 (10 μg/ml) for 15 minutes. B) Effects of SR-0379 on phosphorylated FAK (Tyr397 and Tyr925) and phosphorylated Akt (Ser473) as determined by Western blot. The cells were treated with SR-0379 (0.1, 0.3, 1, 3 and 10 μg/ml) for 30 minutes. C, D) Effects of RGD peptide and wortmannin on the SR-0379-induced phosphorylation of FAK (Tyr397 and Tyr925) and Akt (Ser473) as determined by Western blot. The cells were preincubated with RGD peptide (30, 100 and 300 μM, an inhibitor of integrin-ligand interactions) (C) or wortmannin (100 nM) (D) for 30 minutes and were then treated with SR-0379 (10 μg/ml) for 30 minutes. E) Effects of RGD, wortmannin and rapamycin on the NHDFs proliferation stimulated by SR-0379. The cells were preincubated with RGD (1000 μM), wortmannin (100 nM) or rapamycin (1 nM) for 2 hours and then were treated with SR-0379 (10 μg/ml). N = 3 per group. **P<0.01 vs. control, ## P<0.01 vs. SR-0379 (10 μg/ml). F) Effects of Akt knockdown using siRNA on the NHDFs proliferation stimulated by SR-0379. The cells were pretreated with Akt si RNA or Control si RNA for 24 hours and then were treated with SR-0379 (1, 3 and 10 μg/ml). N = 4 per group. **P<0.01 vs. control si RNA, # P<0.05 vs. SR-0379 (1 μg/ml) treated with control siRNA.

What are the molecular mechanisms of these actions of SR-0379? The phosphorylation effects of SR-0379 on FAK at Tyr397 and Tyr925 and on Akt at Ser473 were examined in NHDFs. As shown in Fig. 3A, treatment with SR-0379 (10 μg/ml) increased the phosphorylation of FAK Tyr397 and Akt Ser473 but not of FAK Tyr925 after 30 minutes, similar to the well-known antimicrobial peptide LL-37. The treatment with SR-0379 at doses of 0.3 to 10 μg/ml also significantly increased the phosphorylation of FAK Tyr397 and Akt Ser473 (Fig. 3B). The involvement of integrins was also tested in NHDFs. Pretreatment with RGD peptide (30, 100 and 300 μM), a small-molecule integrin antagonist, inhibited the activation of FAK and Akt induced by SR-0379 (Fig. 3C). Wortmannin (100 nM) and Rapamycin (1 nM) also inhibited the activation of Akt induced by SR-0379 (Fig. 3D, 3E). The treatment with SR-0379 (10 μg/ml) resulted in a significant increase in cell proliferation of fibroblast, whereas Akt knockdown using siRNA attenuated SR-0379-induced cell proliferation (Figure 3F and Figure S4A in File S1). Similarly, an inhibitor of Akt by Akt inhibitor IV (1 μM) also attenuated SR-0379-induced cell proliferation (Figure S4B in File S1). These results demonstrated the importance of Akt pathway in the effect of SR-0379.

### Acceleration of wound healing by SR-0379 *in vivo*

**Figure 4:**
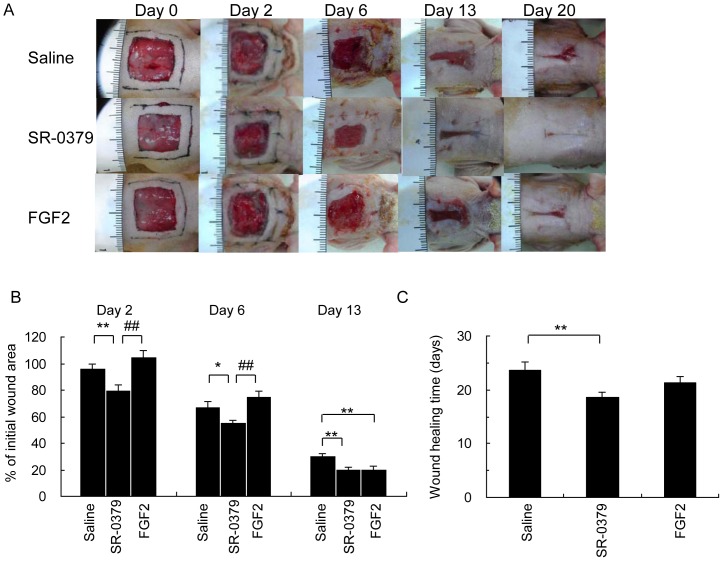
Effects of SR-0379 and FGF2 on full-thickness wound model with flap in diabetic rat model. A) Representative pictures of skin flaps in the streptozotocin-induced diabetic model in the saline (control), SR-0379 (0.2 mg/ml) and FGF2 groups (0.06 mg/ml) on days 0, 6, 13 and 20. B) Quantification of the wound area is represented as a percentage of the initial wound area. N = 6 per group. **P<0.01 vs. control, ##P<0.01 vs. FGF2. C) Days to complete healing by the contraction of full-thickness skin flaps in the streptozotocin-induced diabetic model.

To evaluate the potential use of SR-0379 in clinical practice, a full-thickness wound model with a skin flap was employed in a streptozotocin-induced diabetic rat model. On day 2, the wound area was quickly and significantly reduced in the SR-0379 (0.2 mg/ml) group but not in the saline and FGF2 groups (Fig. 4A, 4B). As shown in Fig. 4A, the color of the wound surface on day 6 was red (bloody) in the SR-0379 treatment group. In contrast, the color of the wound surface was still dark with necrotic skin visible in both the saline and FGF2 groups. Reduced wound area in the SR-0379 group was sustained on days 6 and 13 (Fig. 4B). The skin wound was completely healed at day 19 with SR-0379 treatment, whereas the wound did not heal until day 24 with saline treatment (Fig. 4C).

**Figure 5:**
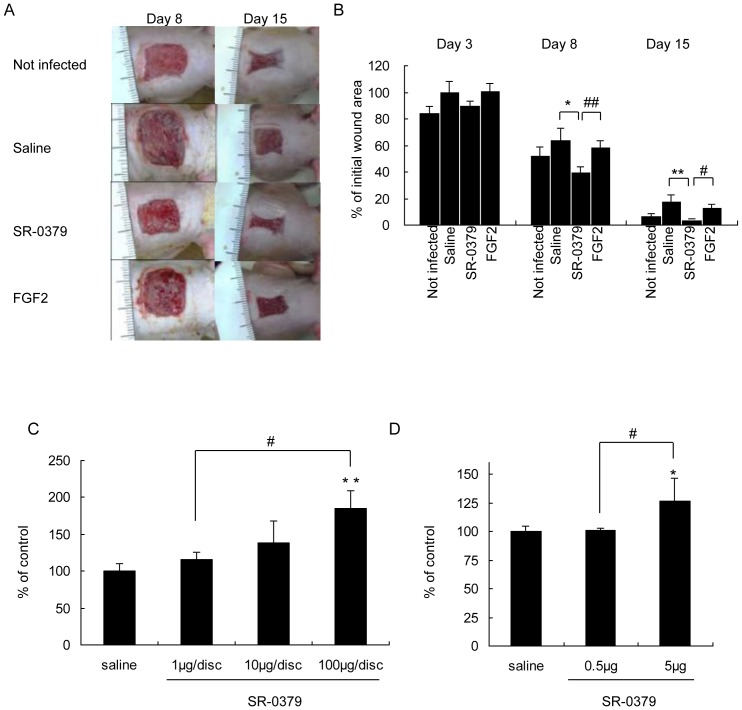
Effects of SR-0379 and FGF2 on the full-thickness skin infected wound model. A) Representative pictures of full-thickness skin flaps in uninfected, saline (control), SR-0379 (1 mg/ml) and FGF2 groups (0.125 mg/ml) on days 8 and 15. B) Quantification of the infected wound area is represented as a percentage of the initial wound area. N = 5 per group. **P<0.01 vs. control, ##P<0.01 vs. FGF2. C) Effects of SR-0379 (1, 10 and 100 μg/disc) on the healing of paper disc implantation in rats. N = 4-5 per group. **P<0.01 vs. control, #P<0.05 vs. 1 μg/disc. D) Effects of SR-0379 (0.5 and 5 μg) on the healing of an experimental open wound in rats. N = 3-4 per group. *P<0.05 vs. control, #P<0.05 vs. 0.5 μg.

To determine the utility of SR-0379 in a clinical situation, we finally evaluated whether the compound could accelerate wound healing in an acute infection wound model, as the presence of infection largely diminishes the wound-healing process. After creating full-thickness defects, *S. aureus* was inoculated. Treatment with SR-0379 (1 mg/ml) significantly reduced the unhealed wound size on days 8 and 15 compared to the saline and FGF2 groups (Fig. 5A, 5B). The effects of SR-0379 on wound healing were more potent than the effects of FGF2, which is currently a standard therapy (Fig. 4B, 5B). To determine the effect of SR-0379 on wound healing, granulation tissue formation was analyzed by subcutaneously implanting a paper disc (Fig. 5C). Granulation tissue weight was significantly increased by treatment with SR-0379 (100 μg/disc). To further evaluate the effect of SR-0379 on wound healing, collagen production and proliferation were measured using the incised wound rat model. The tensile strength after SR-0379 treatment (5 μg) was significantly increased compared to saline treatment. Treatment with SR-0379 induced granulation tissue formation and collagen production, which may accelerate wound healing.

Collagen gel assay is a conventional method to investigate cell behavior that more closely resembles cell behavior *in vivo*. As shown in Figure 2D, the diameter of the collagen gel containing went down by treatment of SR-0379. The promotion of the wound healing with SR-0379 was supported by the enhanced contraction *in vitro*.

## Discussion

Recently, wet dressing has been strongly recommended to accelerate the process of wound healing because epithelial cells and dermal fibroblasts proliferate well under wet conditions [13]. However, wounds under wet conditions present an ideal environment for bacterial growth due to the presence of moisture and warmth. Wound infection has become one of the major risk factors in the delay of wound healing [14], although physicians take many precautions to prevent wound infection. Wound colonization is defined as the presence of multiplying microorganisms on the surface of a wound but with no associated clinical signs and symptoms of infection [15]. Recently, the term critical colonization has gained acceptance and is defined as the borderline of colonization and infection. As wound colonization is most frequently polymicrobial and involves numerous microorganisms that are potentially pathogenic, the diagnosis of clinical colonization is difficult for general physicians in a clinical setting. Recombinant growth factor proteins, such as FGF2 and PDGF, have been shown to stimulate ulcer-healing processes in clinical applications [16], [17]. However, the wound might fail to be healed by these growth factors in the event of an infection, as these growth factors have no antibacterial properties. Therefore, a wound-healing drug with antibacterial properties would be ideal to avoid the risk of infection during wound care.

Based on this idea, we developed AG30, an antibacterial peptide with angiogenic activity. Although our previous study demonstrated that AG30/5C is useful to in the treatment of ulcers [8], [9], cost and stability are problems for clinical applications. Therefore, in this study, we modified AG30/5C as a lead compound to achieve 1) more stability upon exposure to serum, 2) lower cost, 3) wider antibacterial activity and 4) rapid wound healing. The initial analysis using the metabolites of AG30/5C revealed that a core sequence of 20 aa (MLKLIFLHRLKRMRKRLKRK; SR-0007) was enough to stimulate human umbilical vein endothelial cells (HUVECs) proliferation and tube formation at a level similar to AG30/5C. This smaller peptide led to a reduced cost of peptide synthesis. In the previous reports, the replacement with the D-form amino acid improved the proteolytic resistance of antimicrobial peptides [18]. For example, the D-amino acid variants of host defense peptide chicken cathelicidin-2 showed enhanced stability in human serum, and fully resistant to proteolysis by trypsin and bacterial proteases. The modifications increase the stability and lower cytotoxicity of the peptides without altering their antimicrobial potency. We also confirmed the degradation by the peptide bond cleavages in N-terminus of SR-0379, and the change from L-lysine to D-lysine (SR-0379) increased the resistance to serum. Importantly, SR-0379 displayed broader antibacterial activity than the original AG30 and SR-0007. The bactericidal action of antimicrobial peptides such as pexiganan is thought to result from irreversible membrane-disruptive damage [19] [20] [21]. Especially, from the mechanisms of antibacterial activity, SR-0379 exhibited the same MIC against drug-resistant strains, such as aminoglycoside-, carbapenem- and fluoroquinolone-resistant *P. aeruginosa* and MRSA and the multidrug-resistant *A. baumannii*. SR-0379 might be useful to prevent infection by these drug-resistant bacteria.

*In vitro* experiments with SR-0379 demonstrated the induction of proliferation, tube formation, migration and contraction. The closure of cutaneous wounds involves three processes: epithelization, connective-tissue deposition and contraction. In particular, contraction is one of the main factors contributing to epidermal wound healing [22]. The fibroblast-collagen matrix contraction model provides a unique way to study mechanisms. Treatment with SR-0379 promoted contraction in this model, which corresponds to wound healing. The stimulatory effect of SR-0379 on the wound healing process was also confirmed by two *in vivo* wound-healing models. Furthermore, SR-0379 was able to induce angiogenesis and granulation tissue formation in the paper disc model and collagen production and proliferation in the incised wound rat model. These results support the potential use of SR-0379 in the wound-healing process. The ulcer model with infection is a unique model that is especially close to a clinical situation. Importantly, SR-0379 treatment resulted in rapid healing without infection compared to FGF2.

Although the multiple functions of antimicrobial peptides are well known, the mechanisms are still unclear. For example, LL-37 is often reported in the analysis of FPR2 (formerly known as FRPL1), the promiscuous Pertussis Toxin (PTX)-sensitive GPCR and the purinergic receptor P2X7 and in the transactivation of epidermal growth factor receptor (EGFR) [3]. The activation of EGFR in epithelial cells, endothelial cells and fibroblasts by LL-37 resulted in activation of the p38 MAPK, ERK1/2 MAPK, NFkB and PI3 kinase pathways. In contrast, although we also examined the contribution of P2X7 receptors to the effect of SR-0379, the specific antagonist of P2X7 (Brilliant Blue G) failed to inhibit the effects of SR-0379 (data not shown). SR-0379 also weakly activated EGFR. Interestingly, SR-0379 strongly activated FAK, while an integrin inhibitor (RGD peptide) blocked the Akt/mTOR pathway. Downstream of FAK, SR-0379 also activated the PI3 kinase-Akt-mTOR pathway. As mTOR is known to regulate cell growth and survival by integrating nutrient and hormonal signals [10], an inhibitor, rapamycin, attenuated the proliferation induced by SR-0379 in human fibroblasts. The treatment of SR-0379 resulted in increase in cell proliferation of fibroblast, whereas Akt knockdown attenuated the SR-0379-induced cell proliferation These results demonstrate the importance of Akt pathway in the effect of SR-0379.

We have successfully produced SR-0379 as a multifunctional (angiogenic and pro-fibrotic), potent antibacterial peptide with a broad spectrum, including aerobes and anaerobes, Gram-positive and Gram-negative species and drug-resistant and drug-sensitive bacteria and fungi. These properties occur via the activation of PI3 kinase-Akt-mTOR signaling and are useful in the stimulation of wound healing under wet conditions. Further modification of SR-0379 should yield an ideal compound for the treatment of diabetic ulcers, burns and other incurable ulcers. Currently, we plan to test SR-0379 in the treatment of patients with MRSA-positive diabetic and ischemic ulcers.

## Materials and Methods

### Analysis of the AG30/5C metabolites using MALDI-TOF/MS

Rat sera were collected from rats. AG30/5C was incubated in pooled rat serum at 37°C. Samples were collected before incubation, after 10 minutes of incubation and after 60 minutes of incubation and were precipitated by the addition of an equivalent amount of acetonitrile containing 0.1% trifluoroacetate. The samples were centrifuged, and the supernatants were purified using ZipTip m-C18 (Millipore, MA). Sample solution was mixed with matrix solution (α-cyano 4-hydroxy cinnamic acid). The measurement sample for MALDI (0.4 μL) was applied on a MALDI target plate and dried, and the sequences of the AG30/5C metabolites were confirmed by MALDI-TOF/MS analysis (4700 Proteomics Analyzer, Applied Biosystems, CA).

### Serum stability assay

*In vitro* stability studies were performed by incubating the peptide with rat or human serum. Human sera (Pool of donors, 5 men and 5 women) were commercially purchased from KAC (Kyoto, Japan), which has been permitted only for experiment. We don't use the human biological specimens without the documented informed consent. Rat sera were collected from rats. The peptide (500 μg/ml) was added to serum (300 μL) and incubated at 37°C. A part of samples (90 μL) were taken, and the proteins were precipitated with acetonitrile containing 0.1% trifluoroacetate (200 μL). The precipitate was separated by centrifugation. The supernatants were analyzed by high-performance liquid chromatography (HPLC).

### Proliferation, tube formation, cell migration and contraction assays

HUVECs, NHDFs and NHEKs were purchased from Kurabo (Osaka, Japan). The endothelial cells were maintained in HuMedia EB2 and the fibroblasts were maintained in Medium 106S. Both media were supplemented with 1% fetal bovine serum (FBS) as described previously [9]. The epidermal keratinocytes were maintained in HuMedia KB2. Cells were incubated at 37°C in a humidified atmosphere of 95% air/5% CO_2_ with exchange of medium every 2 days. HUVECs were cultured in 96-well plates at a density of 10,000 cells/well and incubated for 48 hours at 37°C with AG30/5C or FGF2 (recombinant human FGF basic, R&D systems, Inc., Minneapolis, MN). The proliferation of HUVECs, NHDFs and NHEKs was analyzed using a WST-1 assay (Dojindo, Kumamoto, Japan). Tubule formation assay has recently been developed in which endothelial cells are co-cultured with fibroblasts. An angiogenesis assay kit (Kurabo, Osaka Japan) was used according to the manufacturer's instructions. Various concentrations of peptides or FGF2 were added to the medium. After 11 days, the cells were incubated with diluted primary antibody (mouse anti-human CD31, 1∶4,000) for 1 hour at 37°C and diluted secondary antibody (goat anti-mouse IgG alkaline phosphatase-conjugated antibody, 1∶500) for 1 hour at 37°C; visualization was achieved with 5-bromo-4-chloro-3-indolyl phosphate/nitro blue tetrazolium (BCIP/NBT). The tube-like structures were measured in terms of total tube length with the software (Angiogenesis Image Analyzer, Kurabo, Osaka Japan). Cell migration was evaluated using an Oris cell migration assay kit (Platypus Technologies, LLC., Madison, WI) according to the manufacturer's instructions. Briefly, the assay utilizes cell-seeding stoppers to restrict cell seeding to the outer annular regions of the wells. Removal of the stoppers reveals a 2-mm diameter unseeded region, the migration zone, into which the seeded cells migrate. The number of cells that migrated into the detection zone was measured using a plate reader. Cellular collagen gel contraction assays were performed as previously described [23] [24]. A solution of collagen and NHDFs (2×10^6^ cells/ml) was added to a 24-well plate at 37°C for 1 hour, and medium supplemented with DMEM containing 10% FBS was then added. The cells were cultured for 24 hours. The culture medium was removed, and DMEM (serum-free) containing SR-0379 or FGF2 was added. The cell-embedded matrix was released from the culture dish surface. At each time point, the lattices were digitally photographed from a fixed distance, and their areas were calculated using image analysis software. In the proliferation assay of fibroblast, RGD peptide, Wotmannin, Akt inhibitor IV and Genistein were obtained from Sigma-Aldrich (St. Louis, MO). Rapamycin was obtained from Funakoshi Co., Ltd.(Tokyo, Japan). Akt siRNA I (#6211) and control siRNA I (#6568) were obtain from Cell Signaling (Boston, MA).

NHDFs were plated at a density of 5000 cells per well in 96-well culture plates in the corresponding culture media without antibiotics one day prior to transfection. Lipofectamine RNAiMAX was purchased from Invitrogen. The lipofectamin (2 μL) was gently added to 100 μL medium and the mixture was incubated for 20 minutes at room temperature. The Akt or Control siRNA was added to the mixture and was incubated for 5 minutes. Transfection complexes were added to each well. NHDFs were incubated for 24 hours at 37°C in a CO_2_ incubator and then SR-0379 (10 μg/ml) was added. NHDFs proliferation was analyzed using a WST-1 assay.

### Measurement of MICs against Bacteria and Fungi

Antimicrobial activity of the peptides was evaluated against *Escherichia coli* JCM 5491, *Pseudomonas aeruginosa* JCM 6119, *Staphylococcus aureus* JCM2874, *Salmonella* Typhimurium JCM1652, *Acinetobacter baumannii* JCM6841, *Bacteroides fragilis* JCM11019, *Fusobacterium nucleatum* JCM11025, *Penicillium glabrum* JCM22534, *Fusarium solani* JCM11383, *Alternaria alternata* JCM5800 (RIKEN, A research institution for basic and applied science in Japan), *Micrococcus luteus* NBRC13867, *Bacillus subtilis* NBRC3134, *Propionibacterium acnes* NBRC107605, *Trichophyton mentagrophytes* NBRC6124, *Trichophyton rubrum* NBRC9185, *Candida krusei* NBRC1395 (National Institute of Technology and Evaluation, Tokyo, Japan), *Salmonella* Enteritidis IID604 (The Institute of Medical Science, The University of Tokyo, Tokyo, Japan). Additionally, the clinical isolates (Drug-sensitive/resistant *Pseudomonas aeruginosa* and *Staphylococcus aureus*, Osaka University Hospital) and multidrug-resistant *Acinetobacter baumannii* (ATCC BAA-1605) were used. The MICs (expressed as μg/ml) of AG30/5C, SR-0007 and SR-0379 were determined by the broth microdilution method as previously described [8], [9]. Serial two-fold dilutions of peptide were added to 0.1 ml of medium containing each type of bacteria and fungi at concentrations of 0.4×10^4^ – 5×10^4^ CFU/ml. The plates were incubated at 37°C with vigorous shaking for 24 or 48 hours. The MICs were determined as the lowest concentrations of peptide that inhibited visible bacterial growth.

### Western blot analysis

Protein extracts (15 μg) were resolved by 10% SDS-PAGE and were then transferred to nitrocellulose membrane. Western blotting was performed. Phospho FAK (Tyr397), Akt, phospho Akt (Ser 473), mTOR, phospho mTOR (Ser2448) and α-Tubulin antibodies were obtained from Cell Signaling (Boston, MA). FAK antibody was obtained from Millipore (Billerica, MA). Phospho FAK (Tyr925) antibody was obtained from Abcam (Cambridge, MA).

### Real-time reverse transcription-polymerase chain reaction (RT-PCR) analysis and ELISA

Expression of the human IL-8 mRNA was measured using real-time reverse transcription polymerase chain reaction (RT-PCR). Total RNA was extracted from the tissue samples using ISOGEN reagent (NIPPON GENE, Toyama, Japan). Complementary DNA (cDNA) was synthesized using the Thermo Script RT-PCR System (Invitrogen, Carlsbad, CA). Relative gene-copy numbers for IL-8 mRNA and glyceraldehyde-3-phosphate dehydrogenase (GAPDH) were determined by real-time RT-PCR using TaqMan Gene Expression Assays (IL-8: Hs00174103_m1; GAPDH: 4352934). Absolute gene-copy numbers were normalized to GAPDH using a standard curve.

The cell free culture supernatants were harvested after treatment of SR-0379 (1, 3 and 10 μg/ml) at 24, 48 and 72 hours. The amount of IL-8 was measured by enzyme-linked immunosorbent assay (ELISA) (R&D Systems, Minneapolis, MN, USA) according to the manufacturer's instructions.

### Effect of SR-0379 on wound healing in a streptozotocin-induced diabetic model

This experimental protocol was approved by the committee for ethics in animal studies of AnGes MG. Male HWY/Slc rats (7 weeks) were given a single intravenous injection of 65 mg/kg streptozotocin (STZ, Sigma-Aldrich, St. Louis, MO), and whole-blood glucose was monitored 24 hours later. This strain is hair less in adult and suitable for wound healing model. The glucose level criterion for diabetes was set at 300 mg/dl. STZ-induced diabetic rats were anesthetized. The square flap (1.73 cm × 1.73 cm) was made in the back of rats. In the center of the flap, the square wound (1.41 cm × 1.41 cm) with full-thickness defect was made (area per wound; 2 cm^2^). In the flap model, skin was cut in three directions of square wound to partially block the blood flow to wound. SR-0379 (0.2 mg/ml, 50 μl), FGF2 (0.06 mg/ml, 50 μl) or saline (control) was administered to each wound (each time point from day 0 to 28). Dressings (Perme-roll, Nitto Denko, Japan) were applied to the wounds. We took a picture of wound with scale every time and calculated the size of scanned image using software (http://hp.vector.co.jp/authors/VA004392/Download.htm#lenara).

### Effect of SR-0379 on wound healing in a cyclophosphamide-induced immunodeficient infection model

Male HWY/Slc rats (7 weeks) were given a single intravenous injection of 100 mg/kg cyclophosphamide (CPA, Wako Pure Chemical Industries, Ltd., Osaka, Japan) and were anesthetized for the preparation of a full-thickness skin flap 24 hours later. CPA-treated rats with white blood cell counts lower than 5,000 were used. The bacteria (*S. aureus*, 1×10^5^ CFU/ml) was applied to each wound on days 0, 1, 2 and 3. SR-0379 (1 mg/ml, 50 μl), FGF2 (125 μg/ml, 50 μl) and saline (control, 50 μl) were administered to the wound at time points on days 0 to 27. Dressings (Perme-roll, Nitto Denko, Japan) were applied to the wounds. Healing size was evaluated by photographing the wound area at a close and fixed distance. The remaining unhealed wound size was measured from the image.

### Evaluation of granulation tissue formation in a paper disc implantation model

Granulation tissue formation was determined as described previously [25]. A paper disc containing saline or SR-0379 (1, 10 and 100 μg) was implanted into the subcutaneous tissue on the backs of 9-week-old Crl:CD(SD) rats under anesthesia. Four or five rats were used for each experimental condition. The paper disc was removed on day 8 and the granulation tissue around the paper disc was weighed after the removal of absorbed fluids with paper wipe.

### Evaluation of collagen production and proliferation in the incised wound rat model

The dermises of Crl:CD(SD) rats (7 weeks) were incised under anesthesia. In the back of rats, we cut the skin (30 mm) and sutured 3 points (Nylon thread, Natsume Seisakusho Co., Ltd., Tokyo, Japan). SR-0379 (0.5 and 5 μg per day) was topically administered in sutured wound one a day for 5 days, and during the period the suture was removed at day 3. At day 6, extracted skin was fixed in one side and pulled in another side. The tension was monitored until the opening of sutured wound. In this evaluation, the increase in tension reflects the strength of sutured wound.

### Statistical analysis

All values are expressed as the means + SEM. Analysis of variance and a subsequent Fisher's Least Significant Difference test were used to determine the significance of differences in multiple comparisons.

## Supporting Information

File S1 **Supporting figures S1–S4.** Figure S1, MALDI-TOF MS analysis. A) Major metabolites of AG30/5C determined by MALDI-TOF MS. Parent compound (AG30/5C) was incubated with rat serum *in vitro* for 10 minutes and 60 minutes. The metabolites were identified by the comparison with that from pre-incubation. Figure S2, Effect of SR-0379 on cell proliferation. Normal Human Epidermal Keratinocytes (NHEKs) were treated with SR-0379 (1, 3 and 10 μg/ml). The results were shown as percent increase compared with control (no treatment). N = 3 per group. *P<0.05 vs. control. Figure S3, Effect of Akt pathway on SR-0379-induced cell proliferation. A) Knockdown of Akt expression by siRNA was confirmed with western blot analysis anti-Akt antibody and anti-α-tubulin antibody. The sample was extracted from NHDFs with no treatment (NT), non-target siRNA (C: control) and Akt siRNA. B) Effects of Akt inhibitor on NHDFs proliferation stimulated by SR-0379. The cells were preincubated with Akt inhibitor IV (1 μM) for 1 hour and then were treated with SR-0379 (1, 3 and 10 μg/ml). N = 3 per group. *P<0.05 vs. control, **P<0.01 vs. control, ## P<0.01 vs. SR-0379 (1 μg/ml), †† P<0.01 vs. SR-0379 (3 μg/ml), ‡‡ P<0.01 vs. SR-0379 (10 μg/ml). Figure S4, Up-regulation of interleukin-8 (IL-8) induced by treatment of SR-0379. A) IL-8 mRNA expression was quantified by real time PCR and shown as a relative expression compared with that of GAPDH mRNA. NHDFs were treated with SR-0379 (10 μg/ml) for 24 hours. Effects of Wortmannin (PI3kinase inhibitor, 100 nM) and Genistein (Tyrosine-specific protein kinase inhibitor, 100 nM) on SR-0379-induced IL-8 mRNA expression. N = 3 per group. *P<0.05 vs. control, **P<0.01 vs. control, ## P<0.01 vs. SR-0379 (no inhibitor). B) IL-8 levels in culture supernatants form NHDF was measured by ELISA at 24, 48 and 72 hours after treatment. NHDFs were treated with SR-0379 (1, 3 and 10 μg/ml) for 72 hours. N = 2.
(PDF)
