# Supplementary material for: Novel Anti-Microbial Peptide SR-0379 Accelerates Wound Healing via the PI3 Kinase/Akt/mTOR Pathway
Source: PLoS One. 2014 Mar 27;9(3):e92597. doi: 10.1371/journal.pone.0092597 (PMC3968008; doi:10.1371/journal.pone.0092597)
Supplement: File S1 — Supporting figures S1–S4. Figure S1, MALDI-TOF MS analysis. A) Major metabolites of AG30/5C determined by MALDI-TOF MS. Parent compound (AG30/5C) was incubated with rat serum in vitro for 10 minutes and 60 minutes. The metabolites were identified by the comparison with that from pre-incubation. Figure S2, Effect of SR-0379 on cell proliferation. Normal Human Epidermal Keratinocytes (NHEKs) were treated with SR-0379 (1, 3 and 10 μg/ml). The results were shown as percent increase compared with control (no treatment). N = 3 per group. *P<0.05 vs. control. Figure S3, Effect of Akt pathway on SR-0379-induced cell proliferation. A) Knockdown of Akt expression by siRNA was confirmed with western blot analysis anti-Akt antibody and anti-α-tubulin antibody. The sample was extracted from NHDFs with no treatment (NT), non-target siRNA (C: control) and Akt siRNA. B) Effects of Akt inhibitor on NHDFs proliferation stimulated by SR-0379. The cells were preincubated with Akt inhibitor IV (1 μM) for 1 hour and then were treated with SR-0379 (1, 3 and 10 μg/ml). N = 3 per group. *P<0.05 vs. control, **P<0.01 vs. control, ## P<0.01 vs. SR-0379 (1 μg/ml), †† P<0.01 vs. SR-0379 (3 μg/ml), ‡‡ P<0.01 vs. SR-0379 (10 μg/ml). Figure S4, Up-regulation of interleukin-8 (IL-8) induced by treatment of SR-0379. A) IL-8 mRNA expression was quantified by real time PCR and shown as a relative expression compared with that of GAPDH mRNA. NHDFs were treated with SR-0379 (10 μg/ml) for 24 hours. Effects of Wortmannin (PI3kinase inhibitor, 100 nM) and Genistein (Tyrosine-specific protein kinase inhibitor, 100 nM) on SR-0379-induced IL-8 mRNA expression. N = 3 per group. *P<0.05 vs. control, **P<0.01 vs. control, ## P<0.01 vs. SR-0379 (no inhibitor). B) IL-8 levels in culture supernatants form NHDF was measured by ELISA at 24, 48 and 72 hours after treatment. NHDFs were treated with SR-0379 (1, 3 and 10 μg/ml) for 72 hours. N = 2. (PDF) [file pone.0092597.s001.pdf]

Figure S1 in File S1

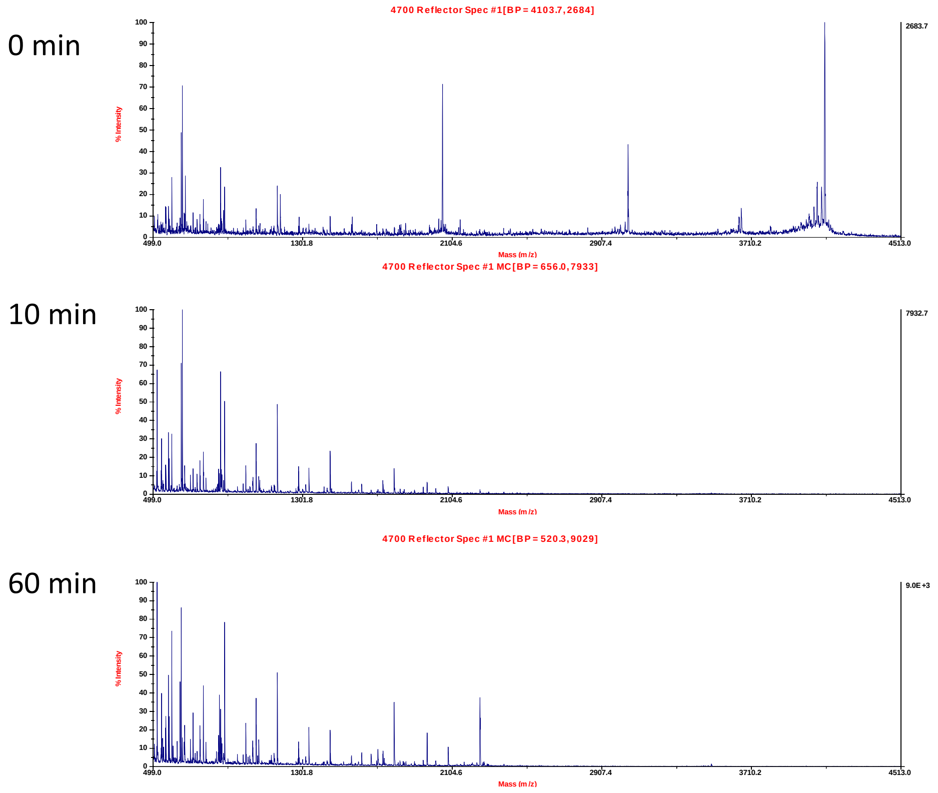

Figure S1 in File S1 MALDI-TOF MS analysis

A) Major metabolites of AG30/5C determined by MALDI-TOF MS. Parent compound (AG30/5C) was incubated with rat serum *in vitro* for 10 minutes and 60 minutes. The metabolites were identified by the comparison with that from pre-incubation.

Figure S2 in File S1

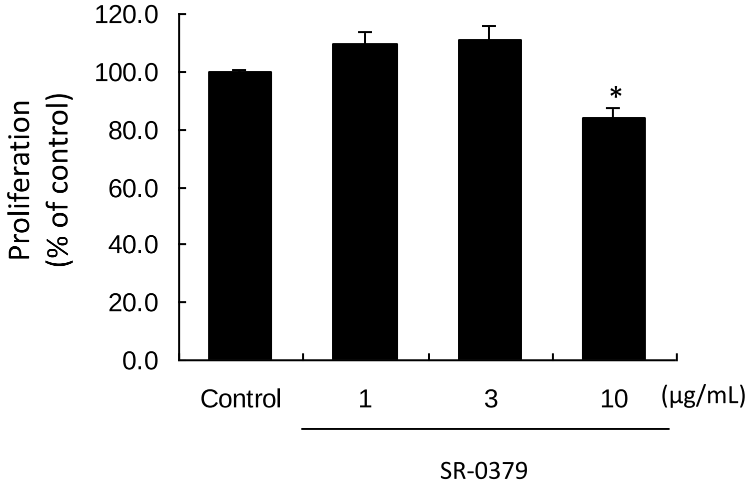

Figure S2 in File S1 Effect of SR-0379 on cell proliferation
Normal Human Epidermal Keratinocytes (NHEKs) were treated with SR-0379 (1, 3 and 10 µg/ml). The results were shown as percent increase compared with control (no treatment). N=3 per group. *P<0.05 vs. control.

Figure S3 in File S1

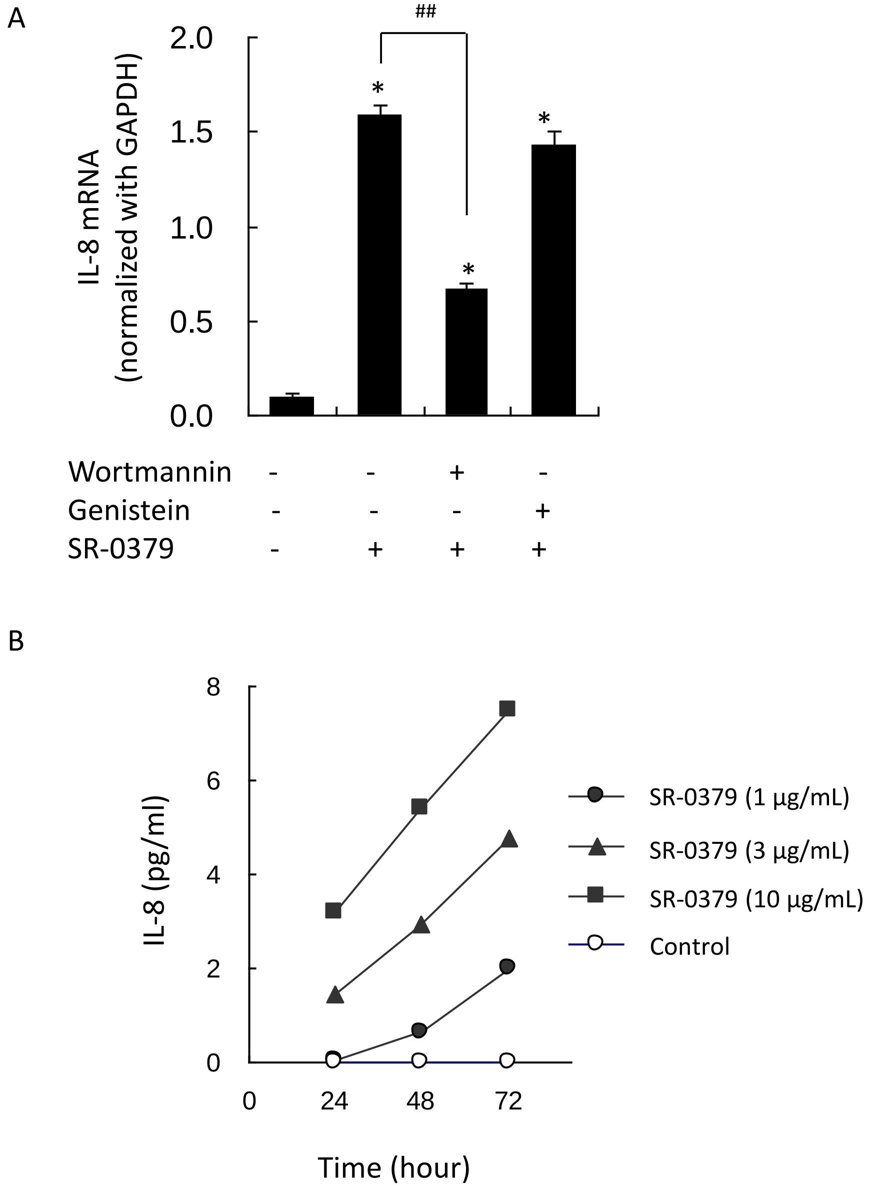

Figure S3 in File S1 Effect of Akt pathway on SR-0379-induced cell proliferation
A) Knockdown of Akt expression by siRNA was confirmed with western blot analysis anti-Akt antibody and anti-α-tubulin antibody. The sample was extracted from NHDFs with no treatment (NT), non-target siRNA (C: control) and Akt siRNA.
B) Effects of Akt inhibitor on NHDFs proliferation stimulated by SR-0379. The cells were preincubated with Akt inhibitor IV (1 μM) for 1 hour and then were treated with SR-0379 (1, 3 and 10 μg/ml). N=3 per group. *P<0.05 vs. control, **P<0.01 vs. control, ## P<0.01 vs. SR-0379 (1 μg/ml), †† P<0.01 vs. SR-0379 (3 μg/ml), ‡‡ P<0.01 vs. SR-0379 (10 μg/ml)

Figure S4 in File S1

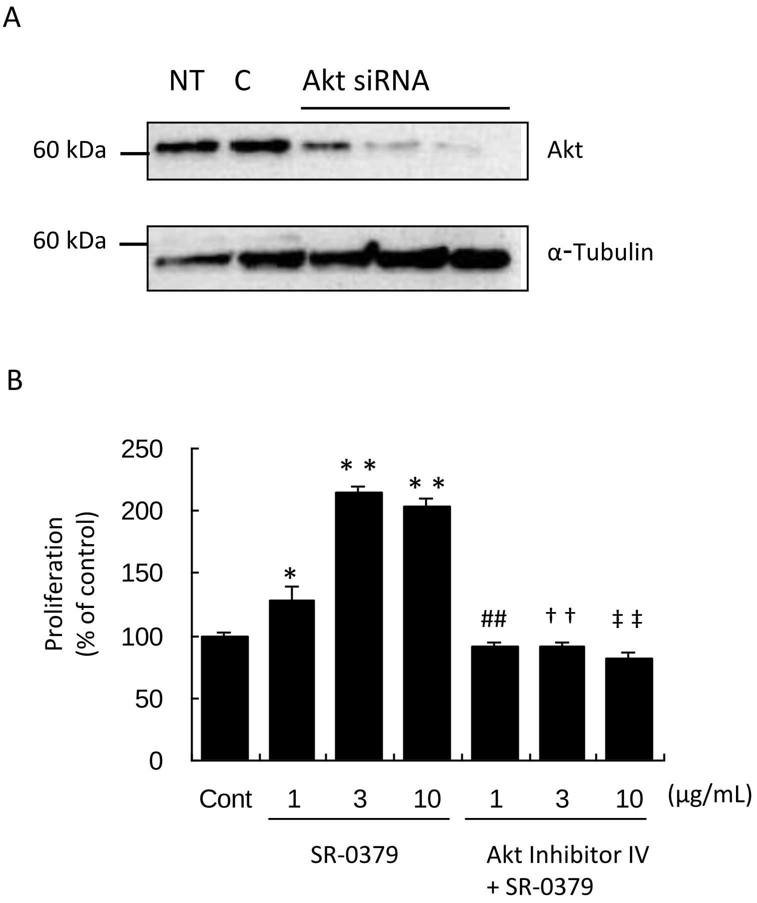

Figure S4 in File S1 Up-regulation of interleukin-8 (IL-8) induced by treatment of SR-0379

A) IL-8 mRNA expression was quantified by real time PCR and shown as a relative expression compared with that of GAPDH mRNA. NHDFs were treated with SR-0379 (10 µg/ml) for 24 hours. Effects of Wortmannin (PI3kinase inhibitor, 100 nM) and Genistein (Tyrosine-specific protein kinase inhibitor, 100 nM) on SR-0379-induced IL-8 mRNA expression. N=3 per group. *P<0.05 vs. control, **P<0.01 vs. control, ## P<0.01 vs. SR-0379 (no inhibitor)

B) IL-8 levels in culture supernatants form NHDF was measured by ELISA at 24, 48 and 72 hours after treatment. NHDFs were treated with SR-0379 (1, 3 and 10 µg/ml) for 72 hours. N=2
